# Amphiregulin Induces iNOS and COX-2 Expression through NF-*κ*B and MAPK Signaling in Hepatic Inflammation

**DOI:** 10.1155/2023/2364121

**Published:** 2023-10-11

**Authors:** Yu Jung Heo, Nami Lee, Sung-E. Choi, Ja Young Jeon, Seung Jin Han, Dae Jung Kim, Yup Kang, Kwan Woo Lee, Hae Jin Kim

**Affiliations:** ^1^Department of Endocrinology and Metabolism, Ajou University School of Medicine, 206, World cup-ro, Yeongtong-gu, Suwon 16499, Republic of Korea; ^2^Institute of Medical Science, Ajou University School of Medicine, Suwon, Republic of Korea; ^3^Department of Physiology, Ajou University School of Medicine, Suwon, Republic of Korea

## Abstract

**Background:**

Inflammation is a major cause of hepatic tissue damage and accelerates the progression of nonalcoholic fatty liver disease (NAFLD). Amphiregulin (AREG), an epidermal growth factor receptor ligand, is associated with human liver cirrhosis and hepatocellular carcinoma. We aimed to investigate the effects of AREG on hepatic inflammation during NAFLD progression, *in vivo* and *in vitro*.

**Methods:**

AREG gene expression was measured in the liver of mice fed a methionine choline-deficient (MCD) diet for 2 weeks. We evaluated inflammatory mediators and signaling pathways in HepG2 cells after stimulation with AREG. Nitric oxide (NO), prostaglandin E2 (PGE2), inducible nitric oxide synthase (iNOS), and cyclooxygenase-2 (COX-2) were analyzed using an enzyme-linked immunosorbent assay and western blotting. Nuclear transcription factor kappa-B (NF-*κ*B) and mitogen-activated protein kinases (MAPKs), including extracellular signal-regulated kinase, c-Jun N-terminal kinase, and p38 mitogen-activated protein kinase, were analyzed using western blotting.

**Results:**

Proinflammatory cytokines (interleukin (IL)-6, IL-1*β*, and IL-8) and immune cell recruitment (as indicated by L3T4, F4/80, and ly6G mRNA expression) increased, and expression of AREG increased in the liver of mice fed the MCD diet. AREG significantly increased the expression of IL-6 and IL-1*β* and the production of NO, PGE2, and IL-8 in HepG2 cells. It also activated the protein expression of iNOS and COX-2. AREG-activated NF-*κ*B and MAPKs signaling, and together with NF-*κ*B and MAPKs inhibitors, AREG significantly reduced the protein expression of iNOS and COX-2.

**Conclusion:**

AREG plays a role in hepatic inflammation by increasing iNOS and COX-2 expression via NF-*κ*B and MAPKs signaling.

## 1. Introduction

Hepatic inflammation is a complex process that occurs in both acute and chronic liver disease [1]. Inflammation plays a key role in liver disease and is a major cause of hepatic tissue damage, promoting the progression of nonalcoholic steatohepatitis, which may eventually advance to cirrhosis and hepatocellular carcinoma (HCC) [2]. As liver damage progresses, immune cells are recruited to the damaged site, and mediators of inflammation, including proinflammatory cytokines, chemokines, and growth factors, initiate and modulate the inflammatory response [3]. The inflammatory response increases the expression of the proinflammatory mediators nitric oxide (NO) and prostaglandin E2 (PGE2), which are regulated by inducible nitric oxide synthase (iNOS) and cyclooxygenase-2 (COX-2) [4, 5].

NO is a proinflammatory mediator that plays an important role in immune regulation, and three NOS isoforms have been identified: neuronal NOS, endothelial NOS, and iNOS [5]. Moreover, iNOS generates high levels of NO under the influence of inflammatory cytokines or other stimuli [6]. Two COX isoforms have also been identified: COX-1 and COX-2. Notably, COX-2 produces large amounts of PGE2 during the inflammatory response [4]. Further, PGE2 is a bioactive lipid that is a crucial mediator of inflammation in disease [7]. Nuclear transcription factor kappa-B (NF-*κ*B) is one of the most important transcription factors for proinflammatory genes and plays a central role in inflammation [8]. The activation of NF-*κ*B controls the activity of numerous genes such as iNOS, COX-2, tumor necrosis factor alpha (TNF-*α*), interleukin (IL)-6, IL-1*β*, and IL-8 in immunity, inflammatory, and stress responses [9]. Mitogen-activated protein kinases (MAPKs) have important functions in regulating cellular responses, contributing to inflammation, cell survival, and cell differentiation [10]. The MAPKs pathway is composed of extracellular signal-regulated kinase (ERK), c-Jun N-terminal kinase (JNK), and p38 mitogen-activated protein kinase (p38-MAPK) [11]. The MAPKs pathway contributes to the regulation of the expression of proinflammatory cytokines, COX-2, and iNOS, and plays an important role in the initiation and maintenance of inflammatory responses [12, 13].

Amphiregulin (AREG) is a ligand of the epidermal growth factor receptor (EGFR). It serves as an autocrine or paracrine growth factor in response to various stimuli, including inflammatory lipids and cytokines [14–16]. Several studies have reported the functional role of AREG in tumorigenesis, such as breast, lung, and HCC [17–19]. According to a recent report, AREG is expressed by multiple populations of activated immune cells in a variety of inflammatory conditions [20]. The association of AREG expression with liver disease has been reported in animal models and human samples. AREG gene expression was detected in human liver cirrhosis and carbon tetrachloride-induced rat cirrhosis [21, 22]. It was recently reported that AREG expression was enhanced in the hepatic stellate cells in both mice fed a methionine choline-deficient (MCD) diet and in severe human nonalcoholic steatohepatitis [23]. Hepatocytes, the main parenchymal cells of the liver, play a pivotal role in liver inflammation [24]. However, to date, the role of AREG and its underlying molecular mechanism in human hepatocytes is still unclear.

Therefore, this study aimed to investigate the role of AREG on hepatic inflammation and related molecular mechanisms using an *in vivo* mouse model of nonalcoholic fatty liver disease (NAFLD) with an MCD diet and hepatocytes *in vitro*.

## 2. Materials and Methods

### 2.1. Materials

Recombinant human AREG was purchased from R&D Systems (Minneapolis, MN, USA). The p38-MAPK inhibitor SB203580 was purchased from Sigma–Aldrich (St. Louis, MO, USA). The JNK inhibitor SP600125 and ERK inhibitor PD98059 were purchased from Selleckchem (Houston, TX, USA). The NF-*κ*B inhibitor Bay 11-7082 was purchased from Calbiochem (San Diego, CA, USA). Antibodies against iNOS, COX-2, JNK, phospho-JNK (T183/Y185), p38-MAPK, phospho-p38-MAPK (Thr180/Tyr182), *α*-tubulin, and Lamin B1 were purchased from Cell Signaling Technology (Danvers, MA, USA). Antibodies against AREG, actin, phospho-ERK (T202/Y204), and ERK were purchased from Santa Cruz Biotechnology, Inc. (Santa Cruz, CA, USA).

### 2.2. Animal Studies

Male C57BL/6J mice (8 weeks old) were purchased from Orient Bio, Inc. (Seoul, Republic of Korea). The animals were housed in a temperature- and humidity-controlled room with a 12 hr light/dark cycle and fed *ad libitum*. After 2 weeks of adaptation, mice were randomly assigned to the normal diet (ND; *n* = 8) and MCD diet (MCD; *n* = 8) groups (*Supplementary 1*). The mice in the ND group were fed a normal chow diet containing 10% fat (D12450B; Research Diets Inc., New Brunswick, NJ) and water. The mice in the MCD group were fed an MCD diet (MCD, A02082002B; Research Diets Inc., New Brunswick, NJ, USA) and water. All treatments and animal care were conducted according to Ajou Institutional Animal Care guidelines and were approved by the Ajou Institutional Animal Care Committee (Permission Number: 2016-0052).

### 2.3. Cell Culture

HepG2 cells were obtained from the American Type Culture Collection and grown in high-glucose Dulbecco's modified Eagle's medium (Welgene Inc., Daegu, Korea) supplemented with 10% (v/v) fetal bovine serum and antibiotics (10 *μ*g/mL streptomycin and 100 IU/mL penicillin) at 37°C in a humidified atmosphere of 95% air and 5% CO_2_ (v/v). Cells were evenly seeded in a 6-well plate at a density of 1 × 10^5^ cells per well for performing experiments.

### 2.4. Measurement of Alanine Aminotransferase and Aspartate Aminotransferase in Plasma

Blood obtained from mouse hearts was immediately centrifuged at 1,500 × *g* for 15 min at 4°C. Plasma was collected and stored at −80°C. Plasma concentrations of alanine aminotransferase and aspartate aminotransferase were measured using the ALT/GPT and AST/GOT kits (Roche Diagnostic International, Mannheim, Germany), respectively, according to the manufacturer's instructions.

### 2.5. Histopathological Staining

Mouse livers were excised, fixed in 4% formalin, and embedded in paraffin. Sectioned tissues were stained with hematoxylin and eosin. Immunohistochemical staining was performed by incubating the tissue sections with a primary antibody against AREG (dilution 1 : 500) in a moisturized chamber at 4°C overnight. The membranes were then incubated with a horseradish peroxidase-conjugated anti-mouse secondary antibody (Dako, Glostrup, Denmark). Stained cells were quantified using ImageJ software (National Institutes of Health, Bethesda, MD, USA).

### 2.6. Enzyme-Linked Immunosorbent Assay (ELISA)

IL-8 concentrations were quantified using the DuoSet kit (R&D Systems) according to the manufacturer's instructions. PGE2 was measured by ELISA (Enzo Life Sciences Inc, Farmingdale, NY, USA) with cell culture medium, according to the protocol provided by the manufacturer.

### 2.7. Measurement of Nitrite Concentration

Nitrite concentrations in the supernatant were determined using a commercially available assay kit based on the radical-mediated conversion of a fluorogenic substrate (OxiSelect In Vitro Nitric Oxide (Nitrite/Nitrate) Assay Kit; Cell Biolabs, San Diego, CA, USA), according to the manufacturer's instructions.

### 2.8. Nuclear Protein Extraction

HepG2 cells were cultured in a 100-mm dish at a density of 4 × 10^6^ cells/dish with or without AREG. Cells were washed twice with PBS, followed by 5 min of incubation in cold 5 mM ethylenediaminetetraacetic acid. Cells were then scraped into 15 mL conical tubes and centrifuged for 5 min at 1,000 rpm. Nuclear extraction was performed using a standard kit according to the manufacturer's protocol (Nuclear Extraction Kit; Abcam, Cambridge, MA).

### 2.9. Western Blot Analysis

RIPA buffer (150 mM NaCl, 1% (v/v) NP-40, 0.5% (w/v) deoxycholate, 0.1% (w/v) sodium dodecyl sulfate, 50 mM Tris–HCl (pH 7.5)), and a protease inhibitor cocktail (pancreatic extract, pronase, thermolysin, chymotrypsin, and papain (Roche Applied Science, Mannheim, Germany)) was used to extract cellular proteins. Equivalent amounts of protein (30 *μ*g) were separated on 4%–12% (w/v) polyacrylamide gels and transferred onto polyvinylidene difluoride membranes (Millipore, Billerica, MA, USA). After blocking with 5% (w/v) skim milk for 30 min, the target antigens were reacted with primary antibodies, followed by addition of secondary antibodies (horseradish peroxidase-conjugated anti-goat IgG or anti-rabbit IgG). Immunoreactive bands were visualized using an enhanced chemiluminescence kit (Amersham Pharmacia Biotech, Piscataway, NJ, USA).

### 2.10. Real-Time Quantitative Reverse Transcription Polymerase Chain Reaction (PCR)

Real-time PCR was performed using the primers listed in Table 1. Total RNA was isolated from mouse liver and HepG2 cells using RNAiso Plus reagent (Takara Bio Inc., Otsu, Japan) according to the manufacturer's instructions. HepG2 cells were evenly seeded in a 12-well plate at a density of 1 × 10^5^ cells per well for performing the experiments. Quantitative real-time PCR was performed using the SYBR Green Master Mix (Takara Bio Inc.) on a Takara TP-815 instrument. All expression levels were normalized to those of *β*-actin.

### 2.11. Statistical Analysis

All experiments were conducted in triplicate, and data are presented as the mean ± standard deviation. The results were analyzed using one-way analysis of variance (ANOVA), followed by Dunnett's multiple comparisons test where appropriate. Significant effects are represented by *P* ≤ 0.05.

## 3. Results

### 3.1. Hepatic Inflammation and AREG Expression Induced in the Livers of Mice Fed with the MCD Diet

**Figure 1:**
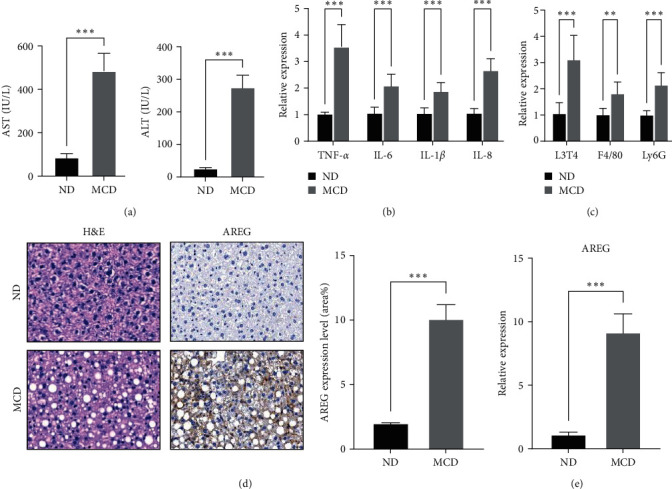
Hepatic inflammation and AREG expression were increased in mice fed an MCD diet for 2 weeks. (a) Levels of alanine aminotransferase and aspartate aminotransferase were evaluated in the plasma of mice fed a normal diet or an MCD diet. (b) The mRNA levels of TNF-*α*, IL-6, IL-1*β*, and IL-8 were measured in the liver by real-time PCR. (c) Expression of L3T4, F4/80, and LyG6 mRNA in the liver was determined using real-time PCR. (d) Immunohistochemical staining with anti-AREG was performed on the livers of mice. Representative images and percentage of AREG + cells. Scale bar: 50 *µ*m. (e) The mRNA level of AREG was measured in the liver by real-time PCR.  ^*∗*^ ^*∗*^ ^*∗*^*P* < 0.001 compared to the ND.

Mice fed with the MCD diet exhibited hepatotoxicity, as evidenced by increased activities of alanine transaminase and aspartate transaminase in the plasma (Figure 1(a)). To verify the inflammatory state of the mice that were fed the MCD diet, we measured the expression levels of genes associated with the hepatic inflammatory response in the liver. The mRNA levels of TNF-*α*, IL-6, IL-1*β*, and IL-8 were significantly higher in the livers of mice fed with the MCD diet than in those who were fed a normal diet (Figure 1(b)). In addition, markers (F4/80, L3T4, and LyG6) for macrophages, T lymphocytes, and neutrophils were highly induced in the mice fed with the MCD diet (Figure 1(c)). Further, hematoxylin and eosin staining showed the presence of lipid droplets in the livers of mice fed with the MCD diet. Elevated levels of AREG in the livers of mice fed with the MCD diet were detected by immunohistochemical staining (Figure 1(d)). Compared to normal control livers, AREG gene expression levels were also upregulated in the livers of mice fed with the MCD diet (Figure 1(e)).

### 3.2. AREG Upregulated Expression of Proinflammation Cytokines and Activated I*κ*B Kinase (IKK)/NF-*κ*B Signaling in HepG2 Cells

**Figure 2:**
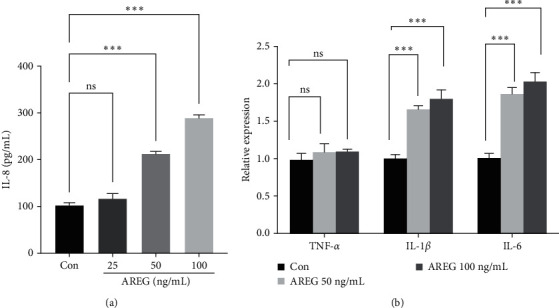
AREG increased the proinflammatory cytokines in HepG2 cells. HepG2 cells were treated with different concentrations of AREG (25–100 ng/mL) for 24 hr. (a) Secretion of IL-8 was determined in cell culture media from HepG2 cells treated with AREG. (b) The mRNA levels of TNF-*α*, IL-1*β*, and IL-6 were measured by real-time PCR  ^*∗*^ ^*∗*^ ^*∗*^*P* < 0.001 compared to the control conditions (Con; no drug).

**Figure 3:**
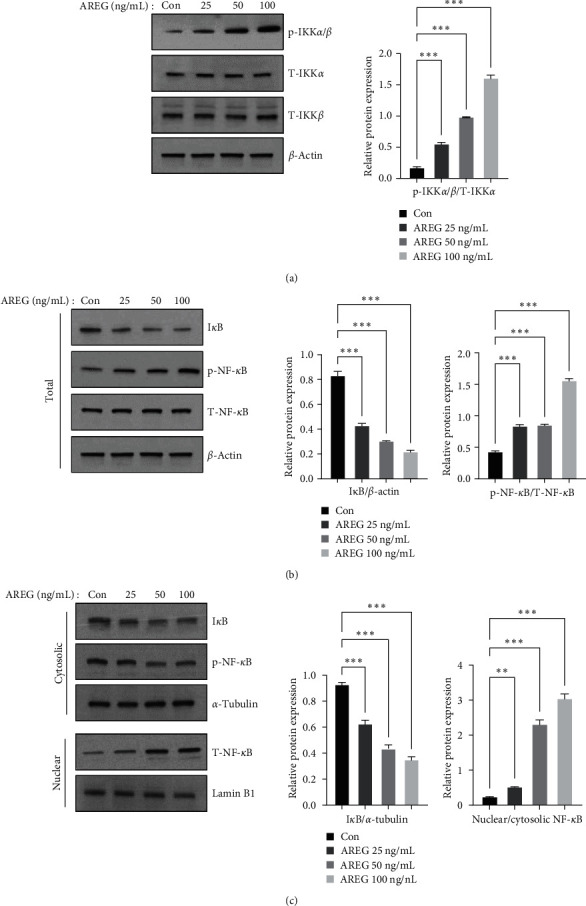
IKK/NF-*κ*B signaling was activated by AREG in HepG2 cells. HepG2 cells were treated with different concentrations of AREG (25–100 ng/mL) for 1 hr. (a) IKK signaling was analyzed using anti-phospho-IKK*α*/*β*, anti-IKK*α*, anti-IKK*α*, and anti-actin antibodies. (b) I*κ*B and NF-*κ*B signaling was analyzed using anti-phospho-NF-*κ*B, anti-NF-*κ*B, anti-I*κ*B, and anti-actin antibodies. (c) The cytoplasmic and nuclear extracts were examined by immunoblotting analysis using anti-NF-*κ*B, anti-I*κ*B, anti-*α*-tubulin, and anti-Lamin B1 antibodies. Graphs represent the ratio between the phosphorylated protein and the total amount of the targeted protein.  ^*∗*^*P* < 0.05,  ^*∗*^ ^*∗*^*P* < 0.01, and  ^*∗*^ ^*∗*^ ^*∗*^*P* < 0.001 compared to the control conditions (no drug).

We hypothesized that AREG triggers an inflammatory response in hepatocytes. We treated HepG2 cells with AREG and measured the levels of proinflammatory cytokines. IL-8 was analyzed in the culture supernatant of AREG-stimulated HepG2 cells by ELISA. Our results showed that AREG increased IL-8 production at concentrations of 50 and 100 ng/mL (Figure 2(a)). The expression of IL-1*β* and IL-6 increased at 50 and 100 ng/mL, but the expression of TNF-*α* did not change (Figure 2(b)). The NF-*κ*B signaling pathway plays a role in the inflammatory response by inducing the transcription of target genes. In an inactive state, NF-*κ*B binds to I*κ*B and activates NF-*κ*B by activating the IKK complex, resulting in phosphorylation of I*κ*B*α* [9]. We used immunoblotting to explore whether AREG activated the IKK/NF-*κ*B pathway. As shown in Figure 3(a), IKK phosphorylation increased in a dose-dependent manner with AREG. The present study further investigated the effect of AREG on the increase in NF-*κ*B nuclear translocation. Upon AREG stimulation, NF-*κ*B translocated into the nucleus (Figure 3(c)); on the contrary, AREG suppressed NF-*κ*B and I*κ*B expression in the cytoplasm (Figures 3(b) and 3(c)).

### 3.3. AREG Activated MAPK-Signaling Pathway in HepG2 Cells

**Figure 4:**
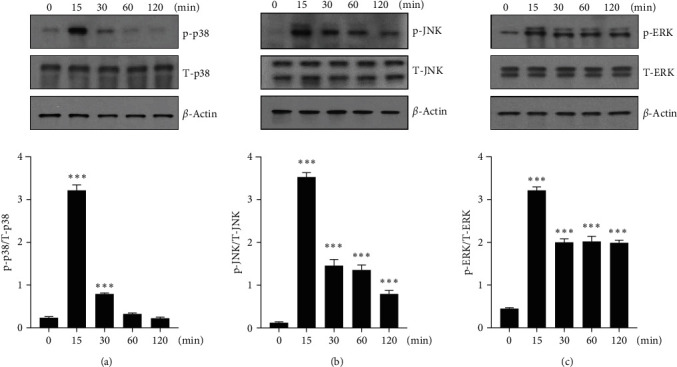
p38-MAPK, JNK, and ERK1/2 signaling were activated by AREG. (a–c) HepG2 cells were incubated with 50 ng/mL AREG for indicated times. Signaling by MAPKs was analyzed using anti-phospho-JNK, anti-phospho-ERK, anti-phospho-p38-MAPK, and anti-actin antibodies. Graphs represent the ratio between the phosphorylated protein and the total amount of the targeted protein.  ^*∗*^*P* < 0.05,  ^*∗*^ ^*∗*^*P* < 0.01, and  ^*∗*^ ^*∗*^ ^*∗*^*P* < 0.001 compared to the time point 0.

The three major MAPKs, p38-MAPK, JNK, and ERK, also play important roles in the inflammatory response [12, 13]. Therefore, we investigated whether AREG was related to MAPKs signaling in HepG2 cells. HepG2 cells were treated with 50 ng/mL AREG for 0, 15, 30, 60, and 120 min, and the effect of AREG on the activation of p38-MAPK, JNK, and ERK1/2 was determined by western blotting. AREG triggered p38-MAPK phosphorylation, which was maximal (3.5-fold) at 15 min (Figure 4(a)). JNK phosphorylation peaked at 15 min (3.5-fold) (Figure 4(b)). ERK phosphorylation peaked at 15 min (3.5-fold increase) (Figure 4(c)). AREG stimulated MAPKs phosphorylation but did not change the levels of total protein or control protein.

### 3.4. AREG Induced COX-2 and iNOS and Increased PGE2 and NO Production in HepG2 Cells

**Figure 5:**
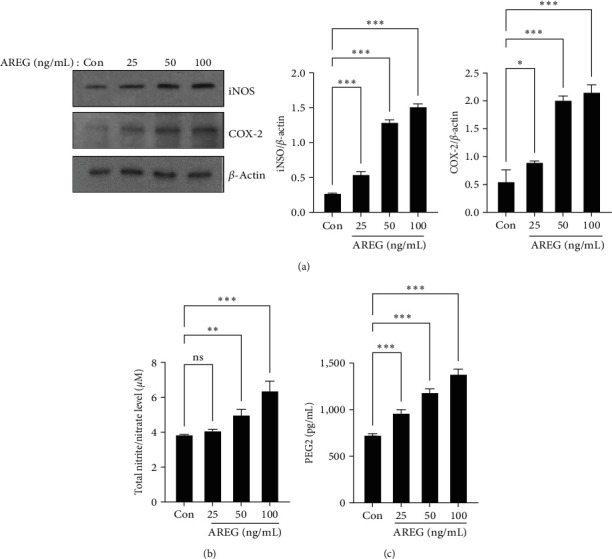
iNOS and COX-2 expression and NO and PGE2 release were increased by AREG in HepG2 cells. HepG2 cells were treated with different concentrations of AREG (25–100 ng/mL) for 24 hr. (a) The expression levels of iNOS and COX-2 were analyzed by western blotting. (b) NO and (c) PGE2 productions were determined in cell culture media from HepG2 cells. Data are expressed as mean ± standard deviation of three independent experiments.  ^*∗*^*P* < 0.05,  ^*∗*^ ^*∗*^*P* < 0.01, and  ^*∗*^ ^*∗*^ ^*∗*^*P* < 0.001 compared with the control conditions (no drug).

The NF-*κ*B and MAPK signaling pathways contribute to the regulation of COX-2 and iNOS expression during the inflammatory response. We measured iNOS and COX-2 after stimulation with AREG in HepG2 cells. AREG increased iNOS expression in a dose-dependent manner in HepG2 cells (Figure 5(a)). NO levels in HepG2 cell supernatants also significantly increased at 24 hr following treatment with 50 and 100 ng/mL (Figure 5(b)). AREG also induced COX-2 expression (Figure 5(a)) and PGE2 production (Figure 5(c)) in HepG2 cells in a dose-dependent manner. Compared to normal control livers, *iNOS* and *COX-2* expression levels were upregulated in the livers of MCD diet-fed mice (*Supplementary 2*).

### 3.5. Inhibition of MAPKs and NF-kB Prevented Expression of iNOS and COX-2 in HepG2 Cells

**Figure 6:**
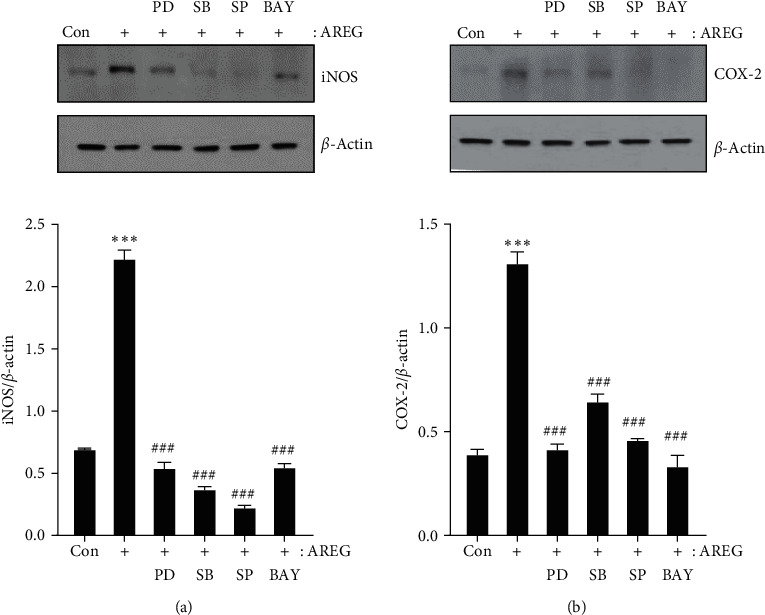
AREG-induced iNOS and COX-2 expression was mediated by NF-*κ*B and MAPKs signaling pathways in HepG2 cells. HepG2 cells were pre-incubated with PD0325901 (10 *μ*M), SB203580 (10 *μ*M), SP600125 (10 *μ*M), and BAY11-7082 (5 *μ*M) for 2 hr before exposure to AREG (50 ng/mL) for 24 hr (a and b). The expression levels of iNOS and COX-2 were analyzed by western blotting.  ^*∗*^ ^*∗*^ ^*∗*^*P* < 0.001 compared with the control conditions (no drug). ^###^*P* < 0.001 compared with the AREG-treated cells.

To further evaluate the potential contribution of signaling pathways in AREG-induced iNOS and COX-2 expression, we identified signaling pathways using selective inhibitors of MAPK-related proteins (ERK inhibitor, PD98059; p38-MAPK inhibitor, SB203580; JNK inhibitor, SP600125) and NF-*κ*B inhibitor (BAY 11-7082) [25]. HepG2 cells were pretreated for 2 hr prior to AREG treatment to block the ERK, p38-MAPK, JNK, and NF-*κ*B signaling pathways and were then treated with AREG, following which changes in iNOS and COX-2 proteins were studied through western blot analysis. Inhibitors of MAPKs and NF-*κ*B efficiently blocked AREG-induced iNOS and COX-2 expression (Figures 6(a) and 6(b)). In addition, MAPK and NF-*κ*B inhibitors suppressed AREG-induced IL-1*β* and IL-6 expression (*Supplementary 3*).

## 4. Discussion

In the present study, we found that AREG expression significantly increased in the liver of the mice modeled for NAFLD, who were fed with the MCD diet. AREG increased the expression of proinflammatory cytokines such as IL-6, IL-1*β*, and IL-8 in HepG2 cells. In addition, AREG significantly induced iNOS and COX-2 and the secretion of NO and PEG2 via the NF-*κ*B and MAPK pathways. NF-*κ*B and MAPK inhibitors blocked AREG-induced iNOS and COX-2 expression in HepG2 cells.

Inflammation is an immune response that is typically present in all disease stages and is associated with the development of fibrosis, cirrhosis, and HCC in the liver [2]. Human samples and mouse models of NAFLD and nonalcoholic steatohepatitis are associated with inflammatory responses in the liver [26, 27]. Although inflammatory responses play a crucial role in NAFLD progression, the mechanisms by which inflammation progresses are not yet understood. AREG was described as a regulator of cell growth factors in breast cancer cells [28]. In other inflammatory responses, AREG is expressed by various activated immune cells such as T cells, macrophages, and neutrophils [15, 29]. Further, AREG is expressed at a high level in the serum of HCC patients [30]. Studies using several human HCC cell lines and tissue samples from patients with primary HCC have shown the overexpression of AREG [31]. In mouse liver fibrosis caused by carbon tetrachloride, Kupffer cells showed higher AREG expression and played an important role in liver fibrogenesis [22]. AREG expression was also enhanced in the hepatic stellate cells of mice fed with the MCD diet and of severe human nonalcoholic steatohepatitis [24].

The MCD diet is a commonly used animal model that produces key features of nonalcoholic steatohepatitis, including steatosis, hepatic inflammation, and fibrosis [32]. Previously, we confirmed that the expression of pro-inflammatory cytokines and activated fibrosis markers were increased in the liver of the MCD-induced NAFLD mouse model after a 4-week diet [33]. Mice fed the MCD diet developed hepatic steatosis starting after 1 week, and inflammation occurred by 2 weeks [34]. In this study, proinflammatory cytokines such as TNF-*α*, IL-6, IL-1*β*, and IL-8 and immune cell recruitment, including T cells, macrophages, and neutrophils were, investigated after 2 weeks of MCD diet. IL-8 has been shown to be involved in inflammatory microenvironment and tumor cell proliferation through activation of EGFR [20]. AREG-induced IL-8 production in a human lung cancer cell line (A549) via pathways involving EGFR, PI3K/AKT, and ERK [35]. We hypothesized that the recruitment of immune cells and the increased expression of IL-8 in the MCD diet model might be related to AREG. Interestingly, AREG expression was increased in immunohistochemistry analysis, and gene expression was significantly increased in the livers of mice fed with the MCD diet.

AREG expression was reported to be induced by multiple immune mediators, including PEG2, cAMP, insulin-like growth factor-1, and transforming growth factor-*β* [29]. In isolated hepatocytes, AREG expression is induced by IL-1*β* and PEG2 but not by hepatocyte growth factor, IL-6, or TNF-*α* [36]. AREG production is suggested to be regulated by the activation of multiple cell types during an immune response. After binding to its ligand, EGFR, AREG promotes several intracellular signaling pathways, including ERK1/2, PI3K/AKT/mTOR, p38-MAPK, and JAK/STAT [37]. Recombinant AREG upregulated the mRNA expression levels of vascular endothelial growth factor, IL-8, and IL-6, but not those of IL-1*β* or TNF-*α* in rheumatoid arthritis fibroblast-like synoviocytes [38]. In animal models of renal fibrosis, AREG inhibition attenuates the expression of inflammatory cytokines and adhesion molecules [39]. In our previous paper, we reported that in a mouse model of NAFLD, induced with a choline-deficient, l-amino acid-defined, high-fat diet, the expression of AREG was significantly increased compared with normal diet [40]. We explored whether AREG is involved in the hepatic inflammatory response using human hepatocytes. Although it has recently been reported that EGFR and AREG silencing increases inflammation and apoptosis in human pulmonary arterial endothelial cells [41], our data showed that AREG upregulated IL-8, IL-1*β*, and IL-6 in HepG2 cells. Different cell types may have different regulatory mechanisms for AREG-induced inflammation.

Previously, we reported that PGE2 production increased significantly in RAW 264.7 macrophages stimulated with lipopolysaccharide compared to cells without lipopolysaccharide induction. COX-2 and iNOS mRNA and protein expression also markedly increased after lipopolysaccharide stimulation [42]. The extreme production of NO related to iNOS synthesis is involved in the inflammatory response [5]. iNOS plays a vital role in releasing NO during the pathophysiology of inflammatory diseases [6]. Additionally, COX-2 is also stimulated by inflammatory stimuli during the inflammatory response [4, 7]. In this study, HepG2 cells stimulated with AREG showed overexpression of NO and PEG2 production compared to the control group. COX-2 and iNOS protein expression also markedly increased after AREG stimulation. These results suggest that AREG is involved in the induction of proinflammatory factors. It was reported that IKK/NF-*κ*B and MAPK signaling pathways are the key pathways for the inflammatory response [8, 12]. Activation of the NF-*κ*B signaling cascade results in the complete degradation of I*κ*B via phosphorylation and ubiquitination [9]. In our study, we observed that p-JNK, p-p38, and p-ERK expression were increased, and there was no significant change in p-NF-*κ*B expression in MCD-fed mice (data not shown). However, in HepG2 cells, AREG increased the phosphorylation of IKK/NF-*κ*B and inhibited I*κ*B. Additionally, we found that AREG also increased the phosphorylation of p38-MAPK, JNK, and ERK in HepG2 cells. Moreover, when HepG2 cells were treated with NF-*κ*B and MAPK inhibitors, we noticed a significant suppression of COX-2 and iNOS protein expression, respectively. This provides evidence that AREG can regulate the expression of COX-2 and iNOS through the NF-*κ*B and MAPK signaling pathways in hepatocytes.

In conclusion, this study suggests that AREG plays a role in hepatic inflammation by increasing iNOS and COX-2 expression via NF-*κ*B and MAPKs signaling. These findings could provide insights into potential therapeutic targets for the treatment of hepatic inflammation.

## Figures and Tables

**Table 1:** Primer sequences used for quantitative real-time-PCR.

Gene	Primer forward	Primer reverse
Human		
TNF-*α*	TGAAAGCATGATCCGGGACG	TGAGGAACAAGCACCGCCTG
IL-1*β*	CCTTTGGTCCCTCCCAGGAA	TGAGTCTGCCCAGTTCCCCA
IL-6	TGTGTGGGGCGGCTACATCT	GCCTTCGGTCCAGTTGCCTT
iNOS	GTCTTGCTTGGGGTCCATCA	GCACATCAAAGCGGCCATAG
COX-2	GAACCTGCAGTTTGCTGTGG	AGAAGCGTTTGCGGTACTCA
Mouse		
TNF-*α*	TCGTAGCAAACCACCAAGTG	AGATAGCAAATCGGCTGACG
IL-6	GACCTGTCTATACCACTTCAC	GTGCATCATCGTTGTTCATAC
IL-1*β*	TCTCGCAGCAGCACATCAACA	CCTGGAAGGTCCACGGGAAA
IL-8	CAAGGCTGGTCCATGCTCC	TGCTATCACTTCCTTTCTGTTGC
L3T4	CCAGCTGTCTGCTTGGATCA	AACATGCGTGTGTTCGTGTG
F4/80	CAACACTCTCGGAAGCTATTAT	GAATTCCTGGAGCACTCATC
Ly6G	TTCCTGCAACACAACTACC	GATGGGAAGGCAGAGATTG
AREG	GAATCGCTTTCTGGGGACCA	ATAGCTGCGAGGATGATGGC

## Data Availability

The data used to support the findings of this study are available from the corresponding authors upon reasonable request.

## Abbreviations

AREG: Amphiregulin
COX-2: Cyclooxygenase-2
EGFR: Epidermal growth factor receptor
ELISA: Enzyme-linked immunosorbent assay
ERK: Extracellular signal-regulated kinase
HCC: Hepatocellular carcinoma
IKK: I*κ*B kinase
iNOS: Inducible nitric oxide synthase
IL: Interleukin
JNK: c-Jun N-terminal kinase
MAPKs: Mitogen-activated protein kinases
MCD: Methionine choline-deficient
NAFLD: Nonalcoholic fatty liver disease
NO: Nitric oxide
NF-Kb: Nuclear transcription factor kappa-B
p38-MAPK: p38 mitogen-activated protein kinase
PRC: Polymerase chain reaction
PGE2: Prostaglandin E2
TNF-*α*: Tumor necrosis factor-*α*.

## Supplementary Materials

Supplementary 1 Time schedule of the animal model.

Supplementary 2 iNOS (a) and COX-2; (b) expression were increased in mice fed an MCD diet for 2 weeks.

Supplementary 3 AREG-induced IL-1*β* and IL-6 expression was blocked by NF-*κ*B and MAPKs signaling inhibitors.
