# Supplementary material for: Amphiregulin Induces iNOS and COX-2 Expression through NF-κB and MAPK Signaling in Hepatic Inflammation
Source: Mediators Inflamm. 2023 Oct 11;2023:2364121. doi: 10.1155/2023/2364121 (PMC10586434; doi:10.1155/2023/2364121)
Supplement: Supplementary 1 — Time schedule of the animal model. [file 2364121.f1.pdf]

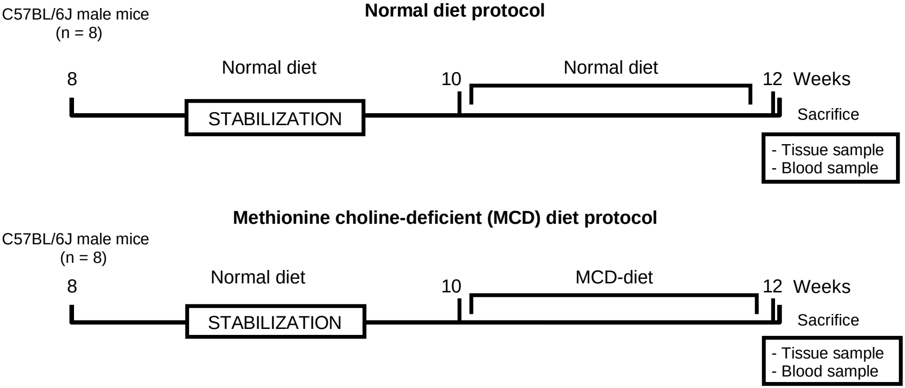
Normal diet protocol
C57BL/6J male mice
(n = 8)
8
Normal diet
STABILIZATION
10
Normal diet
12
Weeks
Sacrifice
- Tissue sample
- Blood sample
Methionine choline-deficient (MCD) diet protocol
C57BL/6J male mice
(n = 8)
8
Normal diet
STABILIZATION
10
MCD-diet
12
Weeks
Sacrifice
- Tissue sample
- Blood sample
