# Supplementary figures and images for: Amphiregulin Induces iNOS and COX-2 Expression through NF-κB and MAPK Signaling in Hepatic Inflammation
Source: Mediators Inflamm. 2023 Oct 11;2023:2364121. doi: 10.1155/2023/2364121 (PMC10586434; doi:10.1155/2023/2364121)

**A**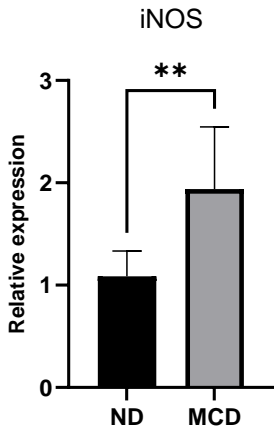**B**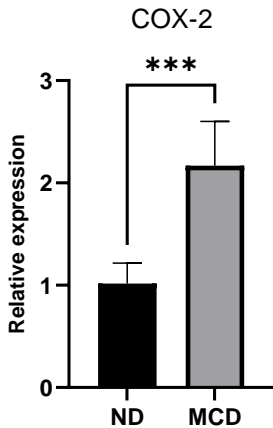

Supplement: Supplementary 2 — iNOS (a) and COX-2; (b) expression were increased in mice fed an MCD diet for 2 weeks. [file 2364121.f2.pdf]

**A**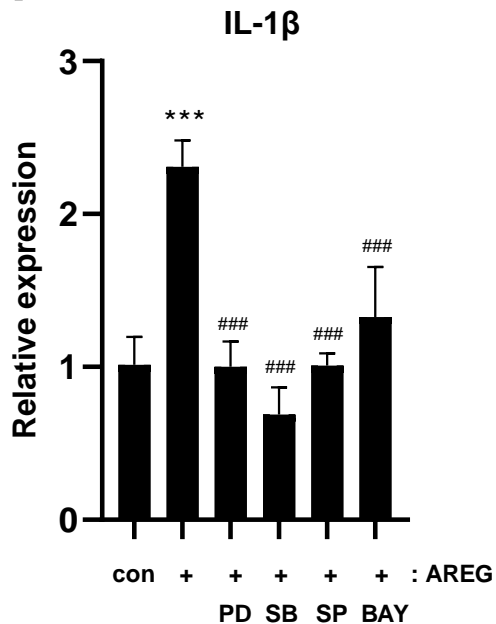**B**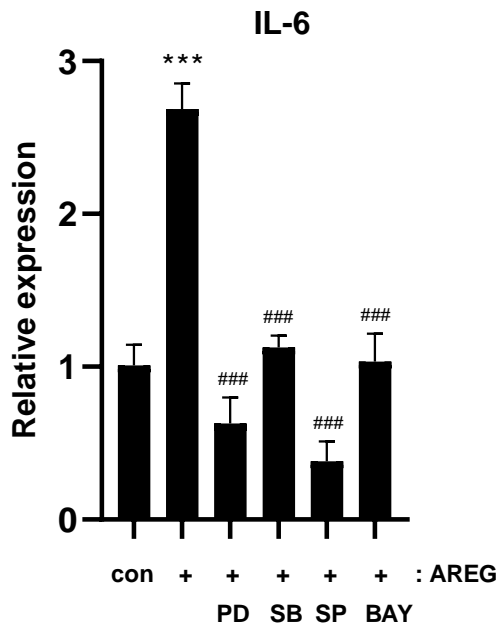

Supplement: Supplementary 3 — AREG-induced IL-1β and IL-6 expression was blocked by NF-κB and MAPKs signaling inhibitors. [file 2364121.f3.pdf]
